# Supplementary material for: A Novel NIR Fluorescent Probe for Highly Selective Detection of Nitroreductase and Hypoxic-Tumor-Cell Imaging
Source: Molecules. 2021 Jul 22;26(15):4425. doi: 10.3390/molecules26154425 (PMC8347683; doi:10.3390/molecules26154425)
Supplement: Supplementary file 1 [file molecules-26-04425-s001.zip › molecules-1267080-supplementary.pdf]

Supporting Information

A NIR Fluorescent Probe for Highly Selective Detection of Nitroreductase and Hypoxic-Tumor-Cell Imaging

Feng Liu [1,2], Hong Zhang [3], Kun Li [3], Yongmei Xie[1], and Zhihui Li [1,2*]

[1] Department of Thyroid Surgery, West China Hospital of Sichuan University, Chengdu, Sichuan 610041, China
[2] Laboratory of Thyroid and Parathyroid Disease, Frontiers Science Center for Disease-related Molecular Network, West China Hospital of Sichuan University, Chengdu, Sichuan 610041, China
[3] College of Chemistry, Sichuan University, Chengdu, Sichuan 610041, China
* Correspondence: Rockoliver163@163.com (Z. Li)

Table of Contents:

1. Material and Instruments

2. Synthesis

3. Fluorescent property of **Py-SiRh-NTR**

4. NMR Spectra of Synthesized Compounds

5. References

Materials and instruments

Unless otherwise noted, all chemical reagents were purchased from commercial suppliers and used without further purification. All solvents were dried according to the standard methods prior to use. In the optical spectroscopic studies, all of the solvents were either HPLC or spectroscopic grade. Thin layer chromatography (TLC) was performed on silica gel plates, and spots were visualized under UV light. Column chromatography was carried out using 200-300 mesh silica gel (Qingdao Ocean Chemicals). NMR spectra were recorded on a Bruker AMX-400 spectrometer at 25°C (^{1}H NMR: 400 MHz, ^{13}C NMR: 101 MHz) and chemical shifts (λ) are expressed in parts per million (ppm) using the internal standard tertramethylsilane or the deuterated solvent ($CDCl_3$, CD_3OD) as reference. Spin multiplicities in ^{1}H NMR are reported as singlet (s), doublet (d), double doublet (dd), double double doublet (ddd), triplet (t), triplet of triplet (tt), multiplet/overlapping peaks (m) or broad (br). The High-resolution mass spectra (HRMS) were obtained on a Finnigan LCQDECA. The pH values were determined by a pH-3c digital pH-meter (Shanghai Lei Ci Device Works, Shanghai, China) with a combined glass-calomel electrode. UV absorption spectra were recorded on a Persee TU-1901 UV-visible spectrophotometer. Fluorescence spectra were measured on a Hitachi F-7000 fluorescence spectrophotometer. Cell imaging was performed on a Zeiss LSM 780 confocal laser scanning microscope.

Cell culture: HepG2 cells were cultured in Dulbecco's modified Eagle's medium (#11965118, DMEM, Thermo Fisher Scientific) supplemented with 10% fetal bovine serum (#26140079, FBS, Thermo Fisher Scientific), penicillin (100 units/ml), and streptomycin (100 μg/ml; #15140163, 10,000 units/ml, Thermo Fisher Scientific) in a 5% CO2 humidified incubator at 37 °C.

Cell staining: For co-localization experiment, 200 nM lyso-Tracker Green (# L7526, LTG, Invitrogen) at 37 °C for 15 min, followed by 2 μM Py-SiRh-NTR for 15 minute. After treatment, the cells were washed 3 times with PBS, and and observed under a confocal laser scanning microscopy (LSM-780, Carl Zeiss, Inc.)

Confocal laser scanning microscopy: The images were ob-tained using an LSM780 confocal laser scanning microscope (Carl Zeiss, Inc.) equipped with a 63×/1.49 numerical aperture oil immersion objective lens and were analyzed with ZEN software (version 2012 SP1, Carl Zeiss, Inc.) and ImageJ soft-ware (version 1.51j8, National Institutes of Health).

Synthesis

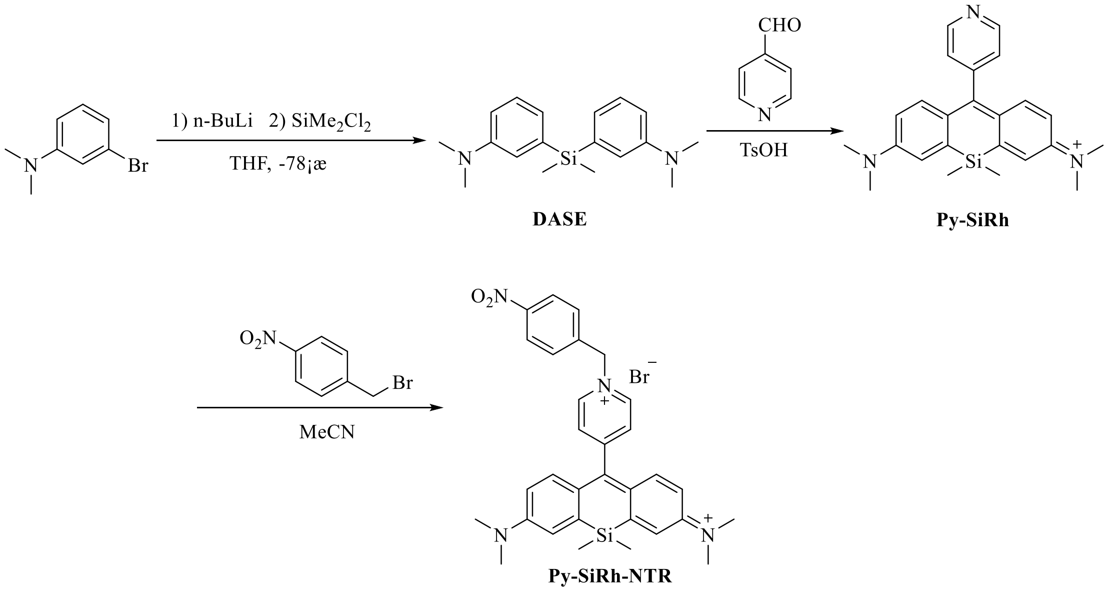

Scheme. S1 Chemical structure and synthetic route of **Py-SiRh-NTR**

Synthesis of diaryl silyl ether (**DASE**)

To a 50 mL well-dried flask flushed with argon, 3-bromo-N, Ndimethylaniline (2.00 g, 10.0 mmol) and diethyl ether (30 mL) were added. The solution was cooled to 0 °C, n-BuLi (1.6 M in n-hexane, 6.88 mL, 11.0 mmol) was added and the reaction mixture was stirred at 0 °C for 2 h. Dichlorodimethylsilane (0.77 mL, 6.0 mmol) dissolved in diethyl ether (10 mL) was added dropwise, and the reaction mixture was slowly warmed to room temperature, then stirred overnight. The reaction was quenched with water (50 mL) and extracted with diethyl ether (50 mL x 3). The organic layers were combined, washed with brine and dried over anhydrous Na_2SO_4. After filtration and removal of the solvent under reduced pressure, the residue was purified by column chromatography on silica gel (petroleum ether: ethyl acetate 30: 1) to afford DASE (1.24 g, 83% yield) as light yellow oil. ^{1} H NMR (400 MHz, CDCl3): 7.28 (t, J = 8.4 Hz, 2H), 6.96 (t, J= 6.8 Hz, 4H), 6.81 (d, J =7.6 Hz, 2H), 2.96 (s, 12H), 0.57 (s, 6H).^{13}C NMR (101 MHz, CDCl3): 150.0, 139.1, 128.6, 122.9, 118.5, 113.7, 40.8, -2.1. ESI (+)-HRMS (m/z): [M]$^{+}$calcd. for: 299.1944, found: 299.1917.

Synthesis of **Py-SiRh**

To a 15 mL sealable pressure tube charged with a magnetic stir bar, the intermediate DASE (0.10 g, 0.34 mmol), compound 4-Pyridinecarboxaldehyde (0.18 g, 1.70 mmol) and p-toluenesulfonic acid monohydrate (0.065 g, 0.34 mmol) were added. The tube was sealed tightly and heated at 140 °C for 12 h. After cooling to room temperature, the

reaction mixture was dissolved in dichloromethane/methanol (5 mL/5 mL). The resulting crude mixture was separated by column chromatography on silica gel (dichloromethane: methanol=80: 1) to give **Py-SiRh** as a blue solid (0.06 g, 45% yield). ^{1} HNMR (400 MHz, CD_3OD): 9.08 (d, J = 6.2 Hz, 2H), 8.12 (d, J = 6.42 Hz, 2H), 7.45 (d, J = 2.58 Hz, 2H), 6.98 (d=9.63 Hz, 2H), 6.83 (dd, J = 2.68 Hz, 2H), 3.38 (s, 12H), 0.63 (s, 6H). ^{13}CNMR (101 MHz, CD_3OD): 159.9, 158.4, 154.3, 147.9, 142.2, 140.5, 128.2, 125.6, 121.9, 114.3, 39.8, -2.5. ESI (+)-HRMS (m/z): $[M]^+$calcd. for: 386.2047, found: 386.2035.

Synthesis of **Py-SiRh-NTR**

SiR-Py (96.5mg, 0.25mmol) and p-Nitrobenzyl-bromide (108mg, 0.5mmol) were dissolved in acetonitrile, and the mixture was refluxed for 24h. The solution was concentrated under vacuum,the resulting crude mixture was separated by silica gel column chromatography (dichloromethane: methanol=30: 1) to give **Py-SiRh-NTR** as a green solid (97mg, 76% yield). ^{1} HNMR (400 MHz, CD_3OD): 8.82(d, J = 6.26 Hz, 2H), 8.23(d, J = 6.15 Hz, 2H), 7.90(d, J = 2.33 Hz, 2H), 7.66(d, J = 2.39 Hz, 1H), 7.18 (d, J = 2.35 Hz, 2H), 6.94 (d, J = 2.31 Hz, 2H), 6.78 (dd, J = 2.55 Hz, 2H), 2.94 (s, 12H), 0.56 (s, 3H), 0.48 (s, 3H).^{13}CNMR (101 MHz, CD_3OD): 170.4, 149.5, 148.6, 144.2, 139.8, 135.5, 131.7, 130.3, 129.9, 124.6, 124.4, 124.0, 115.4, 115.2, 82.9, 39.1, -1.6
ESI (+)-HRMS (m/z): $[M]^+$calcd. for: 522.2440, found: 522.2446.

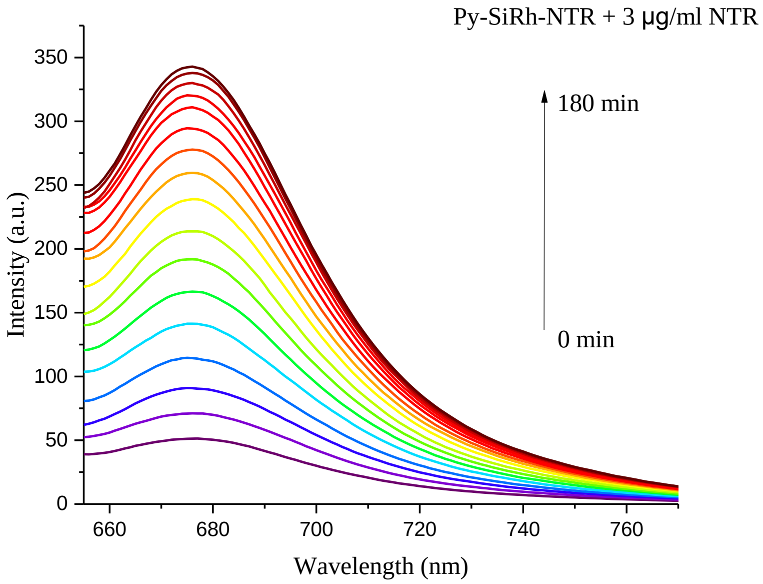

Fig.S1 Fluorescence emission spectra recorded for 10 μM solution of **Py-SiRh-NTR** upon incubating with NTR (3 μg/ml) for varying time intervals (0−180 min) at 37°C in PBS.

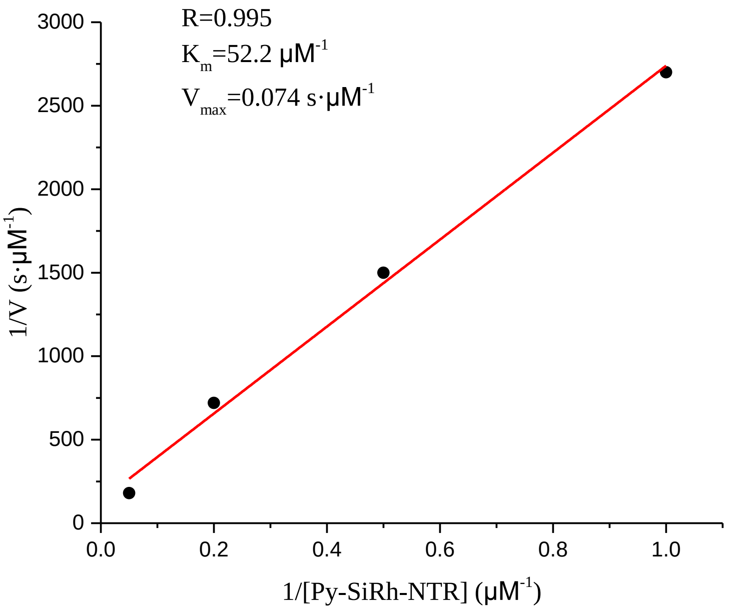

Fig.S2 Lineweaver-Burk plot for the enzyme-catalyzed reaction. The Michaelis-Menten equation was described as: V = Vmax [probe] / (Km + [probe]), where V is the reaction rate, [probe] is the probe concentration (substrate), and Km is the Michaelis constant. Conditions 5.00 μg/mL nitroreductase, 200 μM NADH, 1, 2, 5 and 20 μM of **Py-SiRh-NTR**. Points were fitted using a linear regression model (correlation coefficient R = 0.995).

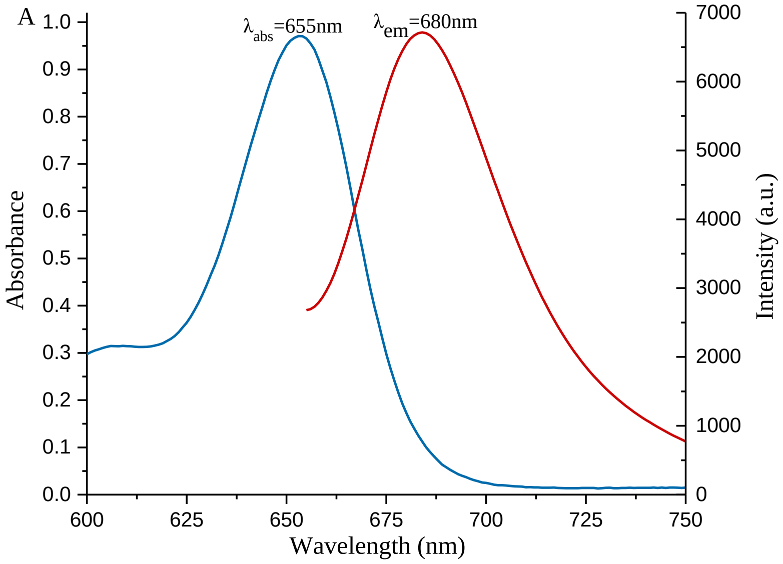

Fig.S3 The normalized absorption spectra and fluorescence emission spectra of 10 μM **Py-SiRh.**

^{1}H, ^{13}C NMR and ESI-MS spectra of synthesized compounds

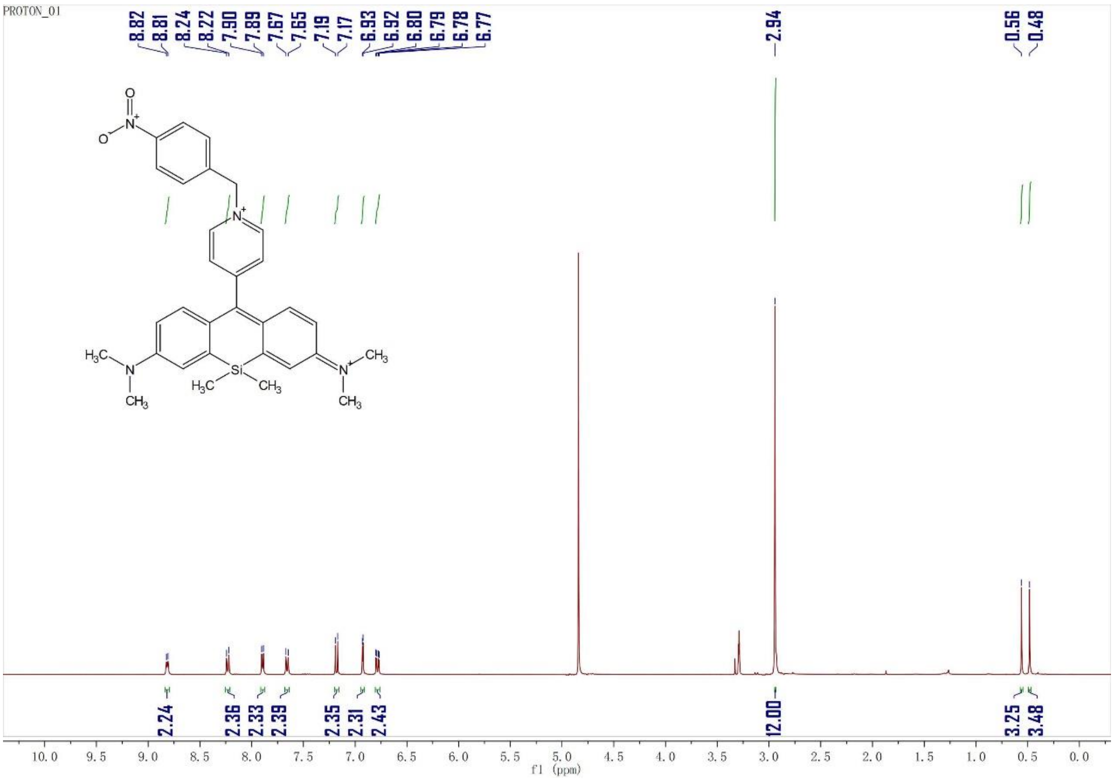

Figure. S4 The ^{1}H NMR spectra of **Py-SiRh-NTR** in CD_3OD.

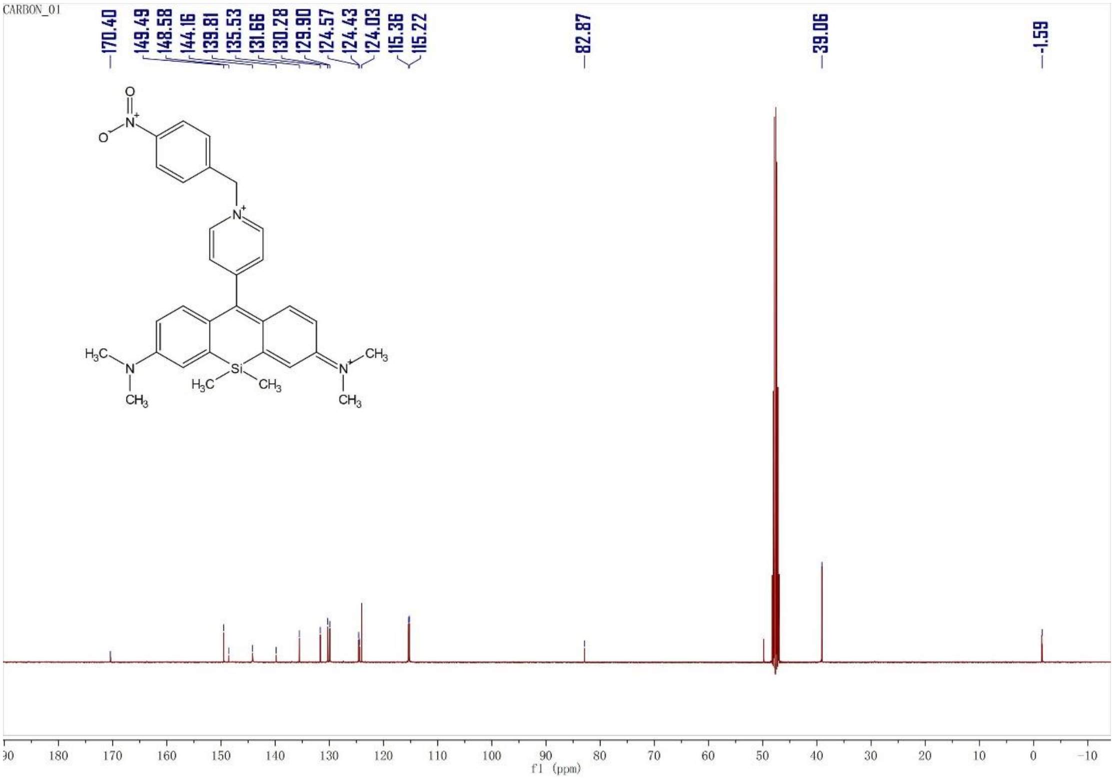

Figure. S5 The ^{13}C NMR spectra of **Py-SiRh-NTR** in CD_3OD.

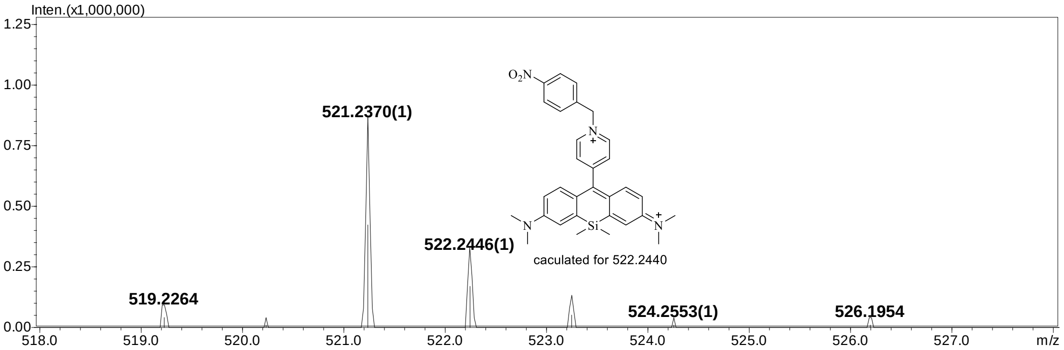

Figre. S6 The ESI-MS spectra of **Py-SiRh-NTR**

References

1. Yu Zhao, Andrea K. Steiger, Michael D. Pluth. *Chem. Commun*., 2018, **54**, 4951-4954
2. Kenjiro Hanaoka, Yu Kagami, Wen Piao, Takuya Myochin, Koji Numasawa, Yugo Kuriki, Takayuki Ikeno, Tasuku Ueno, Toru Komatsu, Takuya Terai, Tetsuo Naganob, Yasuteru Urano *Chem. Commun*., 2018, **54**, 6939-6942
